# Aggregate Impact of Anomalous Noise Events on the WASN-Based Computation of Road Traffic Noise Levels in Urban and Suburban Environments

**DOI:** 10.3390/s20030609

**Published:** 2020-01-22

**Authors:** Francesc Alías, Ferran Orga, Rosa Ma Alsina-Pagès, Joan Claudi Socoró

**Affiliations:** GTM—Grup de recerca en Tecnologies Mèdia, La Salle—Universitat Ramon Llull. c/Quatre Camins, 30, 08022 Barcelona, Spain; ferran.orga@salle.url.edu (F.O.); rosamaria.alsina@salle.url.edu (R.M.A.-P.); joanclaudi.socoro@salle.url.edu (J.C.S.)

**Keywords:** road traffic noise, noise monitoring, dynamic noise maps, anomalous noise events, individual impact, aggregate impact, WASN, sensor nodes, urban and suburban environments

## Abstract

Environmental noise can be defined as the accumulation of noise pollution caused by sounds generated by outdoor human activities, Road Traffic Noise (RTN) being the main source in urban and suburban areas. To address the negative effects of environmental noise on public health, the European Environmental Noise Directive requires EU member states to tailor noise maps and define the corresponding action plans every five years for major agglomerations and key infrastructures. Noise maps have been hitherto created from expert-based measurements, after cleaning the recorded acoustic data of undesired acoustic events, or Anomalous Noise Events (ANEs). In recent years, Wireless Acoustic Sensor Networks (WASNs) have become an alternative. However, most of the proposals focus on measuring global noise levels without taking into account the presence of ANEs. The LIFE DYNAMAP project has developed a WASN-based dynamic noise mapping system to analyze the acoustic impact of road infrastructures in real time based solely on RTN levels. After studying the bias caused by individual ANEs on the computation of the A-weighted equivalent noise levels through an expert-based dataset obtained before installing the sensor networks, this work evaluates the aggregate impact of the ANEs on the RTN measurements in a real-operation environment. To that effect, 304 h and 20 min of labeled acoustic data collected through the two WASNs deployed in both pilot areas have been analyzed, computing the individual and aggregate impacts of ANEs for each sensor location and impact range (low, medium and high) for a 5 min integration time. The study shows the regular occurrence of ANEs when monitoring RTN levels in both acoustic environments, which are especially common in the urban area. Moreover, the results reveal that the aggregate contribution of low- and medium-impact ANEs can become as critical as the presence of high-impact individual ANEs, thus highlighting the importance of their automatic removal to obtain reliable WASN-based RTN maps in real-operation environments.

## 1. Introduction

Environmental noise can be defined as the accumulation of noise pollution caused by sounds generated by human activity outdoors, mainly produced by transport, road traffic, rail traffic, air traffic and industrial activities [1]. According to the World Health Organization, noise exposure produces a loss of around one million healthy life years in Western Europe every year due to different types of derived diseases [2,3]. Focusing on this public health problem, the European (EU) authorities published the Environmental Noise Directive (END) [1] in 2002, which requires the EU member states to tailor noise maps and to develop the subsequent action plans to mitigate noise every five years for major agglomerations and key infrastructures [4]. To address this issue in a harmonized manner, the Common Noise Assessment Methods in Europe (CNOSSOS-EU) was also developed, defining the measurement guidelines to allow comparable noise assessments across the EU [5]. However, as one of the first set of results obtained after the implementation of the END regulation showed [6], noise pollution continues to be one of the principal causes of health problems in Europe. This premise was further endorsed by [7,8], which led to the development of an updated version of the CNOSSOS-EU [9].

The aforementioned dramatic effects of noise pollution on citizens are mainly caused by traffic noise, as it is the main noise source in urban and suburban areas [10,11]. Road Traffic Noise (RTN) maps have been historically created from expert-based measurements using certified devices during specific time periods and locations, considering vehicle flows averaged over long periods of time [12]. During the recordings, the presence of acoustic events non-related to road traffic (e.g., sirens, horns, works, dogs’ barks, airplanes flyovers, etc.) may occur [13]. As a consequence, the collected acoustic data should be cleaned of these undesired events before feeding the noise map creation software [13] to avoid biasing the computation of the A-weighted equivalent sound levels ($L_{Aeq}$) beyond 2 dB, as recommended by the European Commission Working Group Assessment of Exposure to Noise (WG-AEN) [14]. In this context, the Signal-to-Noise Ratio (SNR) of these acoustic events becomes a crucial parameter to evaluate and model [15,16]. Although some researchers have opted to control the SNR of the events by creating artificially mixed datasets (see e.g., [17,18,19,20]), their accurate characterization remains as an open research question as it is almost unfeasible to represent the wide diversity of acoustic data for real world [21].

The so-called Wireless Acoustic Sensor Networks (WASNs) have become an alternative to the creation of noise maps using real-life data, since they allow the ubiquitous monitoring of environmental noise [22,23,24]. During the last decade, several WASNs have been deployed in different smart cities such as Barcelona [25], Algemesí [26], Pisa [27], Monza [28], Halifax [29] and Milan and Rome [30] in Europe, or New York city [31], to name a few. In this WASN-based approach, the traditional manual cleaning of the Anomalous Noise Events (ANEs) on the noise pattern [32] becomes unfeasible due to the huge volume of data that have to be processed in real time [13]. As a consequence, the first generation of these WASN-based environmental noise monitoring systems have mainly been focused on measuring the global sound levels of the sensed locations, without considering the impact of the presence of specific acoustic events on the $L_{Aeq}$ computation. To address this issue, some projects have started incorporating acoustic event detection techniques within the WASN-based noise monitoring pipeline. The Sounds of New York City (SONYC) project includes the real-time identification of 10 common classes of urban sound sources [31] through a machine listening system trained after artificially mixing the events with background noise in the UrbanSound dataset [16]. Moreover, the DYNAMAP project aims at developing a WASN-based dynamic noise mapping system to monitor the acoustic impact of road infrastructures through the creation of noise maps in real time [30]. The project includes two pilot areas: one in the District 9 of Milan as urban area [33], and another in the A90 highway surrounding Rome as a suburban area [34,35]. As the system focuses on measuring RTN levels solely, the ANEs present in the acoustic environments should be automatically removed. To that effect, a machine listening algorithm denoted as Anomalous Noise Events Detector [36] was designed and initially trained using a 9-h expert-based dataset collected from the two pilot areas before installing both sensor networks [21]. The analysis of that preliminary dataset highlighted the importance of the removal of individual ANEs based on their duration and SNR [37]. However, no evidence of a critical impact was yet observed in that dataset due to the presence of several ANEs within the same period of time, probably because the expert-based dataset missed several key aspects from real operation, such as different RTN patterns between day-night and weekday-weekends, or variable weather conditions, among others [38].

After the deployment of the two WASNs in the urban and suburban pilot areas, this paper evaluates the aggregate impact of ANEs on the $L_{Aeq}$ computation of RTN in both environments in real operation. Besides analyzing the individual impact of ANEs on the measurements, the analysis methodology focuses on evaluating the bias caused by the presence of several ANEs within a given period of time, taking into account their impact range (low, medium or high) and sensor location. The study is conducted on 304 h and 20 min of WASN-based labeled acoustic data collected through both sensor networks, before proceeding to update the ANED algorithm with both WASN-based datasets (see [39,40] for a detailed description of the general characteristics of the urban and suburban datasets, respectively).

The paper is structured as follows. Section 2 reviews practices in acoustic environments where the salience and the impact of the events is a key issue. Section 3 presents the impact analysis methodology and impact-related measurements. Section 4 presents the conducted experiments and the results obtained from the analysis of the WASN-based urban and suburban acoustic datasets. Finally, after discussing several key aspects of this work in Section 5, the main conclusions and future work are described in Section 6.

## 2. Related Work

In this section, we review several works from the literature dealing with the identification of salient acoustic events regardless of the noise source; this issue together with the duration of the event sets the basis for the evaluation of the actual impact of these events on the $L_{Aeq}$ computation.

One of the most challenging issues when working with environmental acoustic data recorded in real-life is their accurate characterization, which is supervised by experts. More precisely, this process deals with the parameterization of the data by means of several representative features, among which are the temporal limits of each sound event—i.e., its *actual* duration—by setting up its start and end boundaries [41,42], and its acoustic salience with respect to the background noise [15,16], i.e., the SNR of the event, which is a key parameter to consider. To properly address this issue, it should be taken into account that the events that need to be detected are usually independent one from each other, and typically present a variable duration and SNR. Furthermore, no temporal correlation can be found among them, which makes the challenge of parameterizing audio events particularly more complex compared to speech or music signal [43]. Consequently, the accurate characterization of environmental sound remains as an open research question in real world environments [21].

To work with a controlled environment, artificially-mixed datasets are usually built taking into account a predefined range of SNRs when mixing the events with the background noise during the dataset process generation. Some examples can be found in Foggia et al. [17], Stowell et al. [18] and Socoró et al. [19] (see [21] for further examples). The measurements of SNRs in audio fragments makes it possible to sort events by their degree of acoustic salience with respect to their environment. Moreover, datasets containing synthetic or artificially modified samples also respond to the need to generate more samples of a particular type of noise that is scarce, which is yet today one of the main limitations of acoustic event detection [44]. The explicit SNR measure can be evaluated by means of a closed set of saliency levels, such as −6 dB, 0 dB or +6 dB, as suggested by Stowell et al. in [18]; the authors also propose to record live scripted monophonic event sequences in acoustic environments under control. Foggia et al. [17] mixes several sounds related to surveillance (e.g., scream, glass breaking and gunshots) with both indoor and outdoor environments with six different levels of SNR (from 5 dB to 30 dB, with a step of 5 dB), after the observation of the occurrences of these events in a real-life environment. Socoró et al. [19] presents a dataset composed of a mixture of sound sources considering road traffic noise plus other type of sound events generated using two different SNRs (+6 dB and +12 dB) in order to assess the performance of an anomalous noise event detector. The original non-traffic-noise related audio fragments were extracted from Freesound (https://freesound.org/) while road traffic noise was recorded in a city ring road in real-life conditions. Nakajima et al. [45] works with a dataset recorded in real operation with several examples of noise sources of interest (e.g., cicadas, outside air conditioner, road traffic noise, and neighborhood noise). The work complements the dataset with artificial mixtures to increase the sound source diversity by means of varying the salience of the events using the SNR of three sound sources in the dataset, adapting the margins from −6 dB to +6 dB depending on the characteristics of the noise source. Finally, in Koizumi et al. [46], the authors conduct an objective evaluation on a synthetic dataset, using an open toy-car-running sound dataset; the dataset includes four types of factory noises, and it was generated by mixing synthetically those audio samples at a SNR = 0 dB, together with the audio files of less than 5-s duration from the Task-2 dataset of DCASE 2018 Challenge [47].

Following a different approach, several research works consider auditory attention when evaluating the impact of sound events on acoustic measurements through the evaluation of their SNR levels, whose focus may vary depending on the domain of application (e.g., noise monitoring or surveillance) or the signal of interest (see [48] and references therein for further details). These works analyze the perceptual relevance of audio events according to human response, as in [15], where De Coensel and Bootteldooren design a salience-based map to simulate the capability of humans to switch the attention among several auditory stimuli along time, considering noise examples of means of transportation. This research approach is focused on the identification of the salient event. However, it ignores both its origin and its relative energy with respect to background noise. Following this approach, Salamon et al. [16] included a perceptually based binary descriptor in their dataset to discriminate whether the event was perceived as the main noise source or in the background of the recording. Afterwards, the dataset was used to evaluate the performance of a sound event classification algorithm, getting better accuracy results on foreground events rather than those perceived in the background. Annotating and evaluating a recorded set of audio files is a very time-consuming task. To address these limitations, Salamon et al. published Scaper [20], whose goal is to conduct soundscape synthesis together with data augmentation given a soundbank, controlling characteristics such as the number and type of events, their timing, duration and SNR with respect to a background sound. The final goal is to ease the dataset generation process but also to ensure that the sets of data evaluated present suitable statistical characteristics for training and test of acoustic event detection algorithms.

Finally, it is worth mentioning that a couple of WASN-based projects have recently incorporated the detection of acoustic events in urban and suburban environments in the environmental noise monitoring pipeline. To that effect, the SONYC project [31] has developed a representative dataset with diverse sounds of interest, using the data gathered from the 56 sensors deployed in different neighborhoods of New York, considering up to 10 different common urban sound sources from the urban soundscape (highly frequent in urban noise complaints). The UrbanSound dataset was created after artificially mixing the events coming from Freesound with the background noise collected in the project [16]. Our team, in the framework of the DYNAMAP project [30] made its first attempt to create an acoustic dataset of the urban and suburban pilot areas (District 9 in Milan and A90 highway surrounding Rome) before the sensors of the two WASN were deployed in those scenarios, by means of an expert-based recording campaign [21]. The analysis of those datasets showed the highly local and unpredictable nature of anomalous noise events, which were manually labeled and used to train the preliminary version of the ANED algorithm [36]. Recently, the deployment of the two WASNs in both pilot areas has led to the generation of a suburban acoustic dataset through the 19-nodes WASN in Rome [40], together with the completion of the first steps of the creation of an urban dataset through the 24-node WASN installed in Milan in real operation [39]. From these two experiences, it can be concluded that the evaluation of the acoustic salience of any environmental acoustic event is relevant in order to improve the accuracy of the derived machine listening approaches [43], an issue that was justified in [37] after evaluating the individual impact of the detected events on the overall equivalent noise level computation considering 9 h of real-life acoustic data collected through an expert-based recording campaign. However, as far as we know, no specific analysis has been conducted to assess to what extent the concentration of ANEs with low SNRs within a period of time may bias the WASN-based computation of the $L_{Aeq}$ measurements.

## 3. Impact Analysis Methodology

**Figure 1:**
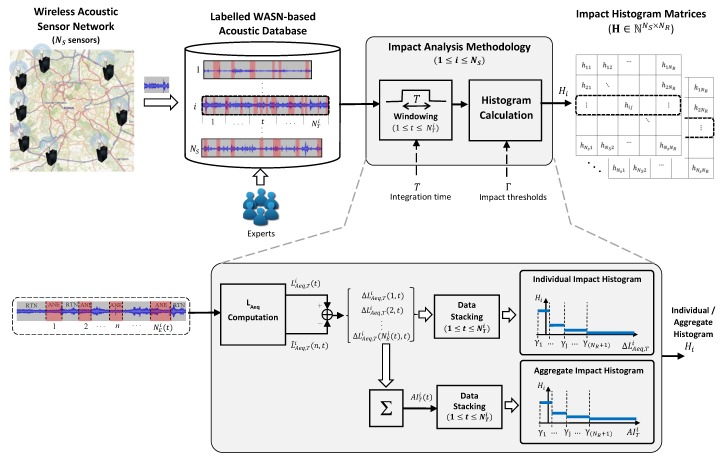
Block diagram of the impact analysis methodology on a labeled WASN-based acoustic dataset obtained from a $N_{S}$ sensors network, where *T* is the integration time considered to compute $L{{}^{i}}_{Aeq,T}\left(t\right)$ and ${\hat{L}}_{Aeq,T}^{i}\left(n,t\right)$ for each sensor *i* and event *n*. Moreover, $\Delta L_{Aeq,T}^{i}\left(n,t\right)$ and $AI_{T}^{i}\left(t\right)$ denote the individual and aggregate impacts of the ANEs, respectively. Finally, $h_{ij}$ represents the components of the histogram matrices H derived from the individual and aggregate impact histograms $H_{i}$, which account for the impact values according to $N_{R}$ impact ranges defined by a set of impact thresholds $\Gamma =\{\gamma_{1},\gamma_{2},\ldots ,\gamma_{\left(N_{R}+1\right)}\}$.

This section describes the methodology followed to analyze the bias caused by ANEs on the $L_{Aeq}$ computation for a given integration time *T* (hereafter denoted as $L_{Aeq,T}$), building on the analysis methodology presented in [37]. The impact analysis methodology permits the study of both individual and aggregate contributions of the anomalous noise events present within a specific period of time. To that effect, individual and aggregate impact histograms are obtained from the labeled data for each sensor of the network according to the considered impact ranges. As depicted in Figure 1, the analysis starts with the labeled acoustic data collected from a WASN of $N_{S}$ sensors in real operation. After windowing the audio streams into frames of *T* seconds, the individual and aggregate impacts of the ANEs present in each period of time *t* are computed and stacked. Finally, both individual and aggregate impact histogram matrices are derived to account for the occurrences belonging to each impact range defined by a set of impact thresholds. The following paragraphs explain the key elements of the proposed analysis methodology in detail.

- **Aggregate impact computation per sensor**
  The *Aggregate Impact* (AI) of several acoustic events can be defined as the accumulated contribution of the individual impacts of all the ANEs present within a period of time and sensor node.
  It is denoted as $AI_{T}^{i}\left(t\right)$, where indexes *i* and *t* respectively represent the sensor number, for $i=\{1,2,\ldots ,N_{S}\}$, and the integration time period, for $t=\{1,2,\ldots ,N_{T}^{i}\}$, $N_{T}^{i}$ being the total number of integration time periods of length *T* considered for its computation given a sensor *i*, and it is defined as
  (1) $$AI_{T}^{i}\left(t\right)=\sum_{n=1}^{N_{E}^{i}\left(t\right)}\Delta L_{Aeq,T}^{i}\left(n,t\right),$$
  where $\Delta L_{Aeq,T}^{i}\left(n,t\right)$ is the individual impact of the *n*-th ANE on the $L_{Aeq,T}$ computation within the integration time period *t*, $N_{E}^{i}\left(t\right)$ being the total number of ANEs present in that time period for sensor *i*, and it is computed as
  (2) $$\Delta L_{Aeq,T}^{i}\left(n,t\right)=L_{Aeq,T}^{i}\left(t\right)-{\hat{L}}_{Aeq,T}^{i}\left(n,t\right),$$
  $L_{Aeq,T}^{i}\left(t\right)$ being the total A-weighted equivalent sound level in the integration period of interest *t* for the *i*-th sensor (i.e., considering RTN and all ANEs found in that *t*), and ${\hat{L}}_{Aeq,T}^{i}\left(n,t\right)$ the corresponding noise level after removing the *n*-th ANE from the measurement through the linear interpolation of the $L_{Aeq,1s}$ values of the previous and subsequent RTN samples (the reader is referred to [37] for further details).
  To that effect, first, the audio data collected from sensor *i* is divided into $N_{T}^{i}$ windows of *T* seconds length (see Figure 1). Next, the A-weighted equivalent noise levels with and without ANEs are computed, whose difference gives the *n*-th individual ANE impact $\Delta L_{Aeq,T}^{i}\left(n,t\right)$. Then, the aggregate impact of window *t* is obtained by accumulating the individual impacts of all the ANEs it contains.
- **Range-based impact analysis per sensor**
  The analysis methodology also aims at categorizing the relevance of both individual and aggregate impacts according to $N_{R}$ impact ranges $\Theta =\{\theta_{1},\theta_{2},\ldots ,\theta_{N_{R}}\}$ (in dB) delimited by a predefined set of impact thresholds $\Gamma =\{\gamma_{1},\gamma_{2},\ldots ,\gamma_{\left(N_{R}+1\right)}\}$, and it is computed as
  (3) $$\Theta =\overset{N_{R}}{\underset{j=1}{⋃}}\theta_{j}=\overset{N_{R}}{\underset{j=1}{⋃}}\left[\gamma_{j},\gamma_{j+1}\right),$$
  where $\theta_{j}$ is defined as the impact range where $\gamma_{j}\leq \Delta L_{Aeq,T}^{i}\left(t\right)<\gamma_{j+1}$, for $j=\{1,2,\ldots ,N_{R}\}$.
  This information is statistically analyzed through the histograms obtained for each sensor (see Figure 1) in the *impact histogram matrix* $H=\left(h_{ij}\right)\in N^{\left(N_{S}\times N_{R}\right)}$, $h_{ij}$ being the number of occurrences of ANEs that account for an impact within $\theta_{j}$ observed in the *i*-th sensor as follows
  (4) $$\begin{array}{c} H=\left(\begin{array}{c} H_{1}\\ H_{2}\\ \vdots \\ H_{i}\\ \vdots \\ H_{N_{S}} \end{array}\right)=\left(\begin{array}{cccccc} h_{11} & h_{12} & \cdots & \cdots & \cdots & h_{1N_{R}}\\ h_{21} & h_{22} & \cdots & \cdots & \cdots & h_{2N_{R}}\\ \vdots & \vdots & \ddots & \ddots & \ddots & \vdots \\ \vdots & \vdots & \ddots & h_{ij} & \ddots & \vdots \\ \vdots & \vdots & \ddots & \ddots & \ddots & \vdots \\ h_{N_{S}1} & h_{N_{S}2} & \cdots & \cdots & \cdots & h_{N_{S}N_{R}} \end{array}\right), \end{array}$$
  where
  (5) $$\begin{array}{c} h_{ij}=\left\{\begin{array}{cc} \sum_{t=1}^{N_{T}^{i}}\sum_{n=1}^{N_{E}\left(t\right)}1_{\theta_{j}}\left(\Delta L_{Aeq,T}^{i}\left(n,t\right)\right) & \mathrm{for}\mathrm{individual}\mathrm{impact},\\ \sum_{t=1}^{N_{T}^{i}}1_{\theta_{j}}\left(AI_{T}^{i}\left(t\right)\right) & \mathrm{for}\mathrm{aggregate}\mathrm{impact}, \end{array}\right. \end{array}$$
  with $1_{\theta_{j}}\left(\cdot \right)$ being the indicator function defined for the interval range $\theta_{j}$ as
  (6) $$\begin{array}{c} 1_{\theta_{j}}\left(x\right)=\left\{\begin{array}{cc} 1 & \mathrm{if}\;x\in \theta_{j},\\ 0 & \mathrm{if}\;x\notin \theta_{j}. \end{array}\right. \end{array}$$
  Notice that rows of H (denoted as $H_{i}$ in Equation (4)) correspond to the impact histograms obtained from each *i* sensor.
- **Analysis of the critical aggregate impacts per impact range and sensor**
  To complement the previous analyses, it is also interesting to identify the origin of critical AIs for those cases that surpass the critical threshold $\gamma_{c}$. To that effect, the aggregate impact of ANEs for a given integration time period and sensor is computed considering only those individual ANEs which $\Delta L_{Aeq}^{i}\left(n,t\right)$ belongs to a particular impact range (i.e., $\Delta L_{Aeq}^{i}\left(n,t\right)\in \theta_{j}$) as follows
  (7) $$AI_{T}^{i}\left(\theta_{j},t\right)=\sum_{n\in \Psi (\theta_{j},t)}\Delta L_{Aeq,T}^{i}\left(n,t\right),$$
  where $\Psi (\theta_{j},t)$ represents the subset of ANE indices within *t* which individual impact belongs to impact range $\theta_{j}$.
  Finally, the *critical AI histogram matrix* $H_{c}=\left(h_{ij}^{c}\right)\in N^{N_{S}\times N_{R}}$ is defined as a particular case of H (see Equation (4)) considering the matrix components as
  (8) $$h_{ij}^{c}=\sum_{t=1}^{N_{T}^{i}}1_{\theta_{c}}\left(AI_{T}^{i}\left(\theta_{j},t\right)\right),$$
  the $1_{\theta_{c}}\left(x\right)$ being a particular case of the indicator function defined by $\theta_{j}=\theta_{c}$ (see Equation (6)), where $\theta_{c}=\left[\gamma_{c},+\infty \right)$ defines the range of critical impacts, as $\gamma_{c}$ represents the threshold of a non-tolerable deviation of the A-weighted equivalent road traffic noise levels.

## 4. Experiments and Results

This section describes the results of the experiments from the impact analysis conducted on the two environmental WASN-based audio databases from the DYNAMAP’s Milan and Rome pilot areas [39,40]. According to the project specifications, the considered integration time to update the $L_{Aeq,T}$ values of the RTN maps is 5 min [30], i.e., T=300 s. To analyze to what extent the collected ANEs from each sensor location bias the $L_{Aeq,300s}$ measurement, the impacts are categorized within three impact ranges (i.e., $N_{R}=3$) [37], accounting for those occurrences (from either individual or aggregate ANEs) causing a low-impact in $\theta_{1}=\left(-\infty ,0.5\right)$ dB, a medium-impact in $\theta_{2}=\left[0.5,2\right)$ dB, and, finally, a high-impact in $\theta_{3}=\left[2,+\infty \right)$ dB, $\theta_{3}=\theta_{c}$ being as this last interval collects those cases that surpass the critical threshold $\gamma_{c}=2$ dB according to the WG-AEN [14]. Regarding the two WASNs, the number of sensors $N_{S}$ considered for the subsequent analyses is 19 for the suburban network, and 23 for the urban one, whereas the total number of evaluated segments of 5 min is 1812 in Milan and 1840 in Rome, respectively.

### 4.1. WASN-Based Environmental Databases

**Figure 2:**
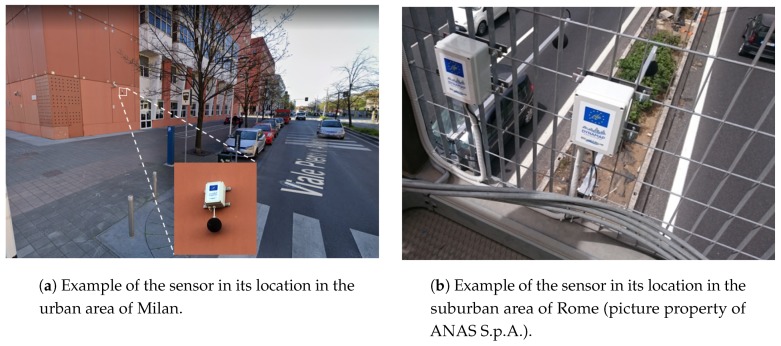
Examples of the location of the low-cost acoustic sensors in the DYNAMAP’s urban and suburban pilot areas.

After the deployment of the sensor networks in the urban and suburban pilot areas of the DYNAMAP project, two WASN-based databases were obtained from environmental acoustic data in real-operation conditions. On the one hand, the nodes distribution across the urban area of Milan is based on the clustering of traffic noise profiles in order to place the best sensor locations for different road categories [33]. On the other hand, in the Rome suburban area, the sensor nodes have been spread along the A90 highway, considering several scenarios of different complexity (single road, crossings, nearby railways and multiple connections) [34,35]. Figure 2 depicts two examples of the sensor placements in both urban and suburban areas, and Appendix A details the sensors’ Ids as well as the description of their locations within Table A1 and Table A2 for the urban and suburban environments, respectively.

In both cases, the recorded databases include data from two days with different traffic conditions: one from a weekday (on Tuesday, the 28th of November 2017 for the urban area, and on Tuesday, the 2nd of November 2017 for the suburban environment), and another during the weekend (on Sunday, the 3rd of December 2017 on the urban area, and on Sunday, the 5th of November 2017 in the suburban environment). The audio recordings were collected in continuous raw audio clips from the first 20 min of each hour (considering a sampling frequency of 48 kHz), as a trade-off between the storage capacity and communications resources of the nodes, and obtaining a representative sub-sampling of the $L_{Aeq}$ measurements along the day [40]. The gathered acoustic data were manually labeled by experts in audio signal processing (see [39,40] for further details). As a result, up to 28 ANE subcategories were identified. Table 1 lists the 16 types of ANEs observed during the manual labeling process in the suburban environment (subcategories being *stru* and *trck* only specifically detected in this scenario), together with the 26 subcategories identified during the annotation of the urban dataset (being *bell*, *blin*, *dog*, *glas*, *peop*, *rubb*, *sqck*, *step*, *tram* and *wrks* those ANE subcategories typically found within this environment). Meteorological-related ANEs like *thun*, *rain* and *wind* cannot be attributed to any specific acoustic environment since they are highly dependent on the weather during the days of the WASN-based data collection. Finally, audio excerpts that contained a mixture of different sound sources (e.g., diverse ANEs together with RTN as background) were labeled as complex sound mixtures or CMPLX. Both CMPLX and ANEs are considered for the subsequent impact-related analyses as both contain undesired acoustic events, after windowing the audio streams into $N_{T}^{i}$ frames of length *T* (see Figure 1).

As a result, the subsequent analyses evaluate 153 h and 20 min of audio data obtained from the 19 sensors placed on the A90 highway portals along the Rome suburban environment, and 151 h obtained from 23 different sensors placed in the building façades of several public buildings across the District 9 of Milan, after discarding node hb114 due to technical problems during the data recording process, but keeping sensor hb119 despite missing some data from the Sunday recordings to $75\%N_{T}^{i}$.

Table 2 summarizes the general characteristics of both analyzed datasets. As can be observed, RTN is the majority class in both cases, as identified 83.7% of the time in the urban environments, while this value raised to 96.5% in the suburban scenario. Accordingly, ANEs were more frequently observed in the urban than in the suburban dataset, being more than four times detected in this environment compared to the suburban one (8.7% of ANE in urban while 1.9% of ANE in suburban). It should be also noticed that the increase of ANE occurrences in the urban environment also fostered the presence of highly complex audio passages.

### 4.2. Individual Impact of ANEs

To understand the relevance of the events, first, a study of the individual ANE impact is conducted following the aforementioned impact analysis methodology. As an overall analysis, Table 3 details the number of occurrences and sensor activation ratios for each environment and recording day.

As can be observed, the presence of anomalous noise events is common in both environments, particularly in Milan which records 10 times more ANEs on Tuesday and 4 times more on Sunday than Rome. Specifically, all recording days have yielded a high percentage of low-impact ANEs, but in Milan, particularly, the presence of low-impact events in relation to the other impact ranges, is higher than in Rome, rising from 98.1 to 99.5% on Tuesday, and from 99.0 to 99.4% on Sunday. In Rome, however, the percentage of medium-impact events is higher than in Milan on both days, with a total of 134 ANEs in Milan and 64 in Rome, respectively. This implies that the sensors in Milan can detect this kind of event in almost all sensors, while only 60% of the sensors in Rome can detect these ANEs. Finally, concerning high-impact events, the percentage of occurrences is similar in both locations, despite Milan has 57 high-impact events detected in 16 sensors and Rome only 12, which activate few sensors.

**Figure 3:**
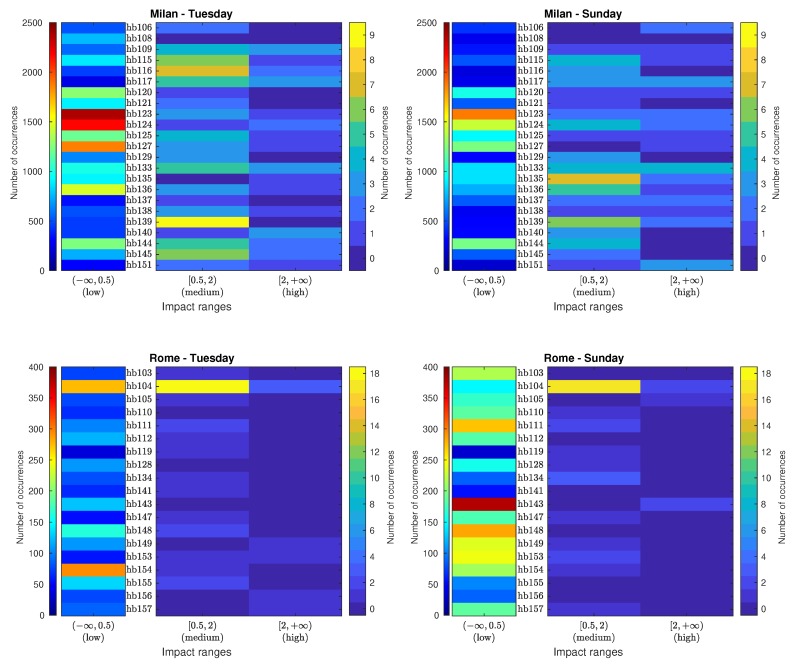
Individual impact histogram matrices (obtained using integration time T=300 s) categorized in three impact ranges (low, medium and high) for the urban (Milan) and suburban (Rome) environments obtained from a weekday (Tuesday) and weekend day (Sunday).

In Figure 3, the corresponding impact histogram matrices for individual ANEs are detailed for each sensor location according to the three impact range intervals (low, medium and high). Notice that the number of occurrences in the low-impact intervals is depicted separately from the medium and high-impact intervals for illustration purposes, as it is more than two orders of magnitude larger.

It can be observed that the maximum number of low-impact ANEs has been found in sensor hb123 of Milan on Tuesday, with 2374 occurrences. In contrast, the maximum number of low-impact events in Rome is 379 for sensor hb143 on Sunday. Concerning the medium-impact events in Milan, the first day accounts for the highest number of events, coming from hb139, which obtains the maximum number of medium-impact ANEs, with 9 occurrences, also presents a significant number in Sunday, with 6 events. In the rest of the cases in Milan, no clear pattern is observed relating both recording days. In Rome, however, sensor hb104 attributes for the maximum number of medium-impact events, with 18 occurrences on Tuesday and 17 on Sunday. This is a particularly relevant case in the suburban area as the second closest sensor is hb134 with only 3 medium-impact events on Sunday. When looking at the column depicting high impact ANEs, it can be observed that a maximum of 5 events were captured on Sunday in sensor hb133 of Milan, while also a significant presence on Tuesday with 4 occurrences. In Rome, sensor hb104 accounts for the highest number of high-impact ANEs on Tuesday, with 3 events, which also recorded one of the highest number of occurrences on Sunday, with 2 events.

### 4.3. Aggregate Impact of ANEs

This section details the results obtained from the analysis of the labeled data in order to find to what extent the presence of several ANEs with low and medium individual impacts within the same integration period can bias the $L_{Aeq,300s}$ computation.

First, Table 4 shows the number of occurrences and sensors activation ratios of the AI for environment and recording day. As it can be observed from the table, the overall presence of occurrences and activation ratios are similar for both days within each location. However, when comparing Milan with Rome, the distribution of the impact ranges differs. In the case of Milan, near 85% of the AIs entail a low impact on the $L_{Aeq300s}$. This percentage increases to almost 96% in Rome. For this reason, the presence, as well the sensor activation, of medium and high-level AIs in Rome is lower than in Milan. In Milan, only one sensor on Tuesday and two on Sunday fail to detect a medium-impact AI. However, in Rome, on Tuesday 7 sensors were not capable of detecting any event and on Sunday the number was 6. In the particular case of high-impact aggregates, their presence is reduced from near 4% in Milan to less than 1% in Rome. Most Milan sensors activate (18 on Tuesday and 17 on Sunday), but only 5 and 3 sensors detect ANEs of this category in Rome in the weekday and during the weekend, respectively.

**Figure 4:**
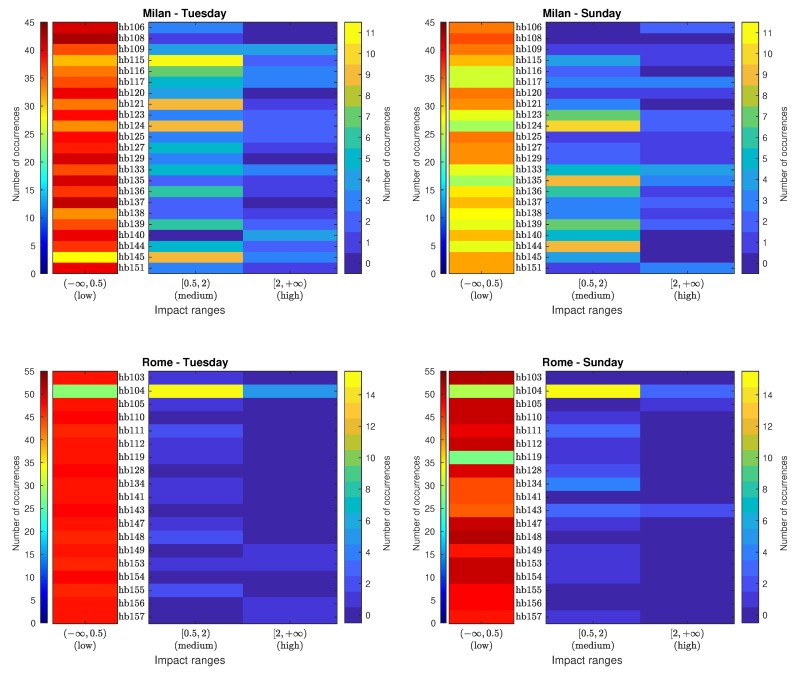
Aggregate impact histogram matrices (obtained using integration time T=300 s) categorized in three impact ranges (low, medium and high) for the urban (Milan) and suburban (Rome) environments obtained from a weekday (Tuesday) and weekend day (Sunday).

Following the same analysis scheme described in the previous section, Figure 4 depicts the AI histogram matrices showing the number of occurrences of aggregate ANEs for each impact range and sensor location for both pilot areas. Again, the number of occurrences in the low-impact range is separated from the rest of occurrences for illustration purposes, due to the same reason indicated in the previous analysis. As can be observed, in Milan, low-impact AIs range from 28 to 43 on Tuesday, and from 24 to 36 on Sunday. A total of 107 intervals on the first day and 88 in the second day contain a medium-impact AI, highlighting sensor hb115 in Milan, with 11 occurrences on Tuesday and hb124 with 10 occurrences on Sunday. However, high-impact AIs record a lower presence of occurrences, with a highest value of 4 in sensors hb109 and hb140 on Tuesday, and in sensor hb133 on Sunday.

Regarding the pilot area in Rome, the presence of low-impact AIs is clearly dominant. However, it is worth mentioning that sensor hb104 presents a completely different pattern, with 15 medium-impact AIs on Tuesday and Sunday. This reduces significantly the low-impact occurrences in that sensor in comparison to other nodes. Finally, as aforementioned, it is to note that sensor hb119 failed in recording several hours of Sunday.

### 4.4. Critical Aggregate Impacts Per Level

**Figure 5:**
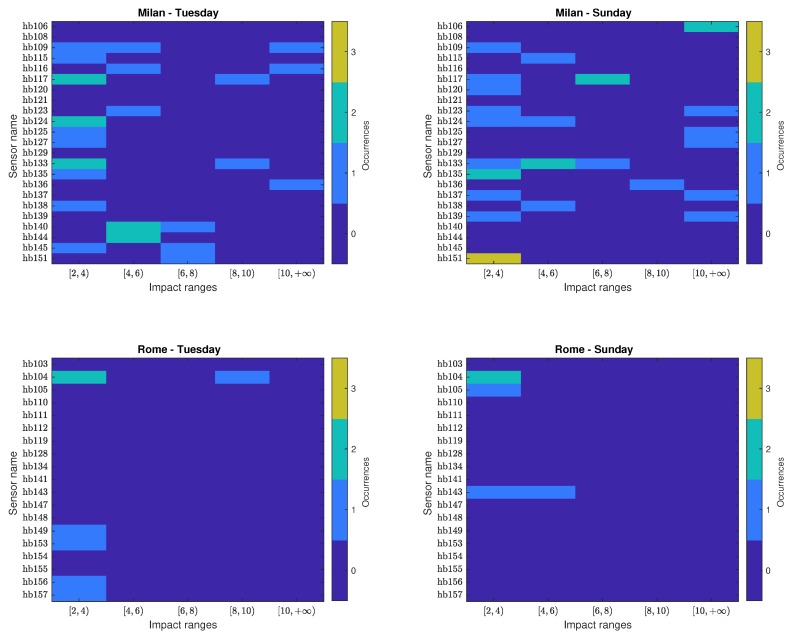
Critical AI histogram matrices ($H_{c}$) of individual ANEs for the urban (Milan) and suburban (Rome) environments obtained from a weekday (Tuesday) and weekend day (Sunday).

In this section, the occurrences that surpass the critical threshold $\gamma_{c}=2$ dB, are analyzed in detail. First, the individual ANEs that bias the $L_{Aeq,300s}$ beyond threshold $\gamma_{c}$ by themselves belong to the high-impact range. To analyze their distribution in detail, the critical individual ANEs observed in Section 4.2 (see Figure 3) are divided in 2-dB spans for each sensor in Figure 5. When analyzing this kind of anomalous noise events, Milan credits for most of the high-impact individual ANEs, most of them within the range of 2 to 4 dB, without belittling their presence in the other ranges for both days. Concerning Rome, sensor hb104 is the one that recorded the largest number of high-impact events, most of them belonging to the [2,4) dB range. Finally, it is to note that 10 events surpass the 10-dB impact range are *sirens*, being the event with the highest impact a 3-min siren with 29.4 dB of impact, recorded in sensor hb137 on Sunday. In contrast, no events surpassing the 10-dB threshold are present in Rome.

**Figure 6:**
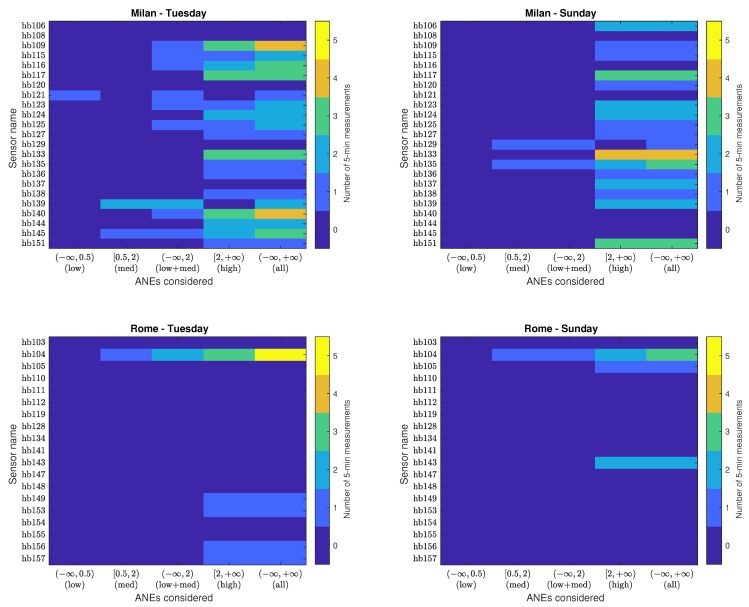
Critical AI histogram matrices ($H_{c}$) categorized in the defined impact ranges for the urban (Milan) and suburban (Rome) environments obtained from a weekday (Tuesday) and weekend day (Sunday).

On the other hand, in order to evaluate if the presence of several ANEs may contribute to the surpassing of the $\gamma_{c}$ threshold, Figure 6 shows the critical AI histogram matrices $H_{c}$ obtained for each network for different impact intervals. That is to say, it depicts the number of times the AI of ANEs contribute to bias the $L_{Aeq,300s}$ of RTN critically for both pilot areas and recording day according to the type of impact range. To that effect, besides considering $\theta_{1}$ (low), $\theta_{2}$ (medium) and $\theta_{3}$ (high) impact intervals to analyze the critical aggregate impacts, two more intervals are considered: $\theta_{1}⋃\theta_{2}$ to account for co-occurring low and medium individual impact ANEs, and $\theta_{1}⋃\theta_{2}⋃\theta_{3}$ to quantify all the critical cases, disregarding the type of the ANE’s individual impact.

The first column of each $H_{c}$ matrices depicted in Figure 6 shows those low-impact AIs causing a critical impact. It can be observed that there is one case accounting for a deviation of the AI higher than 2 dB for a particular period of time *t* of 5 minutes in sensor hb121 installed in Milan. It is due to 13 *wrks* sounds recorded on Tuesday ranging from 0.01 dB to 0.4 dB, i.e., all of them belong to the individual low-impact range $\theta_{1}$, but due to their co-occurrence within the same period of time their AI becomes critical.

Likewise, the second column plots critical medium-impact AIs. In Milan, the threshold $\gamma_{c}$ is surpassed three times on Tuesday and twice on Sunday, whereas in Rome, purely medium-impact occurrences cause a critical AI once each day. Specifically, sensor hb139 collected two of these pieces of evidence on Tuesday. In the first case, the two most significant ANEs are *horns*, with individual impacts of 0.8 and 1.2 dB, respectively (the third one is a *dog* bark with an impact of 0.03 dB). The second is composed of a *horn* of 1.3 dB and two CMPLX sounds, consisting on a mix of RTN and an undetermined beep noise of 0.8 and 0.5 dB. Moreover, sensor hb145 also recorded a period in which individual ANEs bias the $L_{Aeq,300s}$ critically on Tuesday, where the most important event is a *tram* passby of 1.5 dB and the second one is a 1.6-dB CMPLX event consisting of a mix of a *tram* passby and *birds* tweeting near the sensor. On Sunday, two of the periods recorded in the urban environment contain a combination of medium-impact ANEs that surpass the threshold: one in sensor hb129, composed of two distant *sirens* mixed with other sounds, and another due two CMPLX sounds in sensor hb135, containing unidentified mechanical sounds. In what concerns Rome, sensor hb104 presents critical impact evidence due the co-occurrence of purely medium-impact events for both week and weekend periods. On Tuesday two *train* passbys of 1.3 and 1.1 dB bias the $L_{Aeq,300s}$ more than 2 dB. On Sunday, the critical bias is caused by the presence of two *horns* of 1.2 and 1.9 dB, respectively.

The third column of the four AI critical matrices of Figure 6 show the number of times $\gamma_{c}$ is surpassed for ANEs when considering low and medium-impact ANEs, i.e., it collects the occurrences of aggregate low-impact ANEs from $\theta_{1}$ and the aggregate medium-impact ANEs from $\theta_{2}$, as well as the the number of times that the critical threshold is surpassed as a result of the combination of the medium- and low-impact events. This last case is only observed during the weekday 6 times in Milan and once at sensor hb104 in Rome. The latter happens on Tuesday and it consists of the sum of several *train* passbys, with the most salient event an impact of 1.9 dB and the other ones of about 0.1 dB. The six cases in Milan have all been found on Tuesday in different sensors: in hb109, three CMPLX sounds have been found that consist of *train* passbys mixed with RTN of 1.8, 0.2 and 0.2 dB; in hb115, a sum of 13 *wrks* sounds with impacts from 0.01 dB to 0.9 dB; in hb116, a 1.9-dB siren co-occurring with a 0.4-dB CMPLX sound of *birds* mixed with RTN; in hb123, an *airp* of 1.9 dB and other *peop* and *brak*-related sound with impacts smaller than 0.02 dB; in hb125, all significant events are *dog* barks, with impacts of 0.9, 0.6, 0.4, 0.3 dB and decreasing; and in hb140, a *siren* of 1.9 dB has been found, jointly with *people*-related sounds of 0.2 dB.

The next column of critical AI matrices presents high-impact ANEs. For the data at hand, the aggregate high-impact occurrences coincide with the number of individual high-impact events depicted in Figure 3 (see also Table 3, where the number of occurrences in this level is quantified).

Finally, the last column of matrices $H_{c}$ shows the critical AI histogram caused by the co-occurrence of ANEs of any individual impact range altogether. If we focus on the last three columns of Figure 6, it can be appreciated that in all cases, the sum of the low and medium-impact ANEs with the high-impact ANEs results in the total number of times the 2 dB threshold is surpassed. This result could have differed in the case that aggregate low and medium ANEs co-occurred with high-impact ANEs. Therefore, Figure 6 clarifies the fact that high-impact events have not co-occurred at the same 5-min interval for the datasets at hand, besides showing there is no situation in our datasets where low and medium impact aggregated surpass $\gamma_{c}$ at the same 5-min slot *t* in which a high-impact ANE occurs.

To summarize, in Milan, the threshold has been surpassed due to low and medium aggregate impacts in 12 of the 69 critical cases, which correspond to 17% of cases. Likewise, in Rome, the ratio is 3 to 15, corresponding to 20% of the critical cases. Therefore, according to these results, it can be stated that the removal of low and medium-impact ANEs becomes as relevant as high-impact events in order to preserve the accuracy of the RTN level measurements in both urban and suburban environments.

## 5. Discussion

This section discusses several relevant aspects related to the results obtained after applying the impact analysis methodology to the two WASN-based datasets collected from the urban and suburban areas. First of all, it is to note that the individual analysis of the impact of each ANE of those co-occurring within the same integration period has been conducted as a baseline study, since the individual view of the impact of acoustic events unrelated to traffic noise is a straightforward but unrealistic approach to the problem at hand. However, this study has been useful to set the basis for the subsequent aggregate analyses. In this sense, it is worth noting that although the datasets have been collected during specific time periods, the analyzed data show the regular presence of anomalous events across all the days and locations in a real-operation context. Specifically, the number of ANEs found in the urban area is seven times greater than in the suburban environment on average (this ratio being ten times on the weekday). In the suburban environment, the weekday pattern is very similar to what is observed in during the weekend, although a larger number of events have been recorded during the weekend, which should be studied in the future with more detail.

In terms of the acoustic categories, it is worth mentioning that 7.7% of the urban WASN-based dataset and 1.6% of the suburban one has been annotated as CMPLX. As aforementioned, the CMPLX acoustic category can be either caused by a mix of RTN and ANEs or by unidentified ANEs by the experts. The conducted analyses have shown that these kinds of acoustic events can also have a significant impact on the $L_{Aeq,300s}$ computation, showing a similar presence in both datasets as the corresponding ANE acoustic category. Therefore, as well as ANEs, CMPLX audio passages should also be removed from the computation of road traffic noise levels to tailor reliable RTN maps.

When comparing the individual and aggregate impact occurrences for low, medium and high-impact ranges, the analyzed environments present a different distribution. In the case of the urban area, a larger number of low-impact events have been recorded than in the suburban environment. However, as far as AIs are concerned, the percentage of low-impact pieces of evidence are lower in the former than in the latter. In addition, medium and high-impact aggregate ANEs have a significant presence in the urban environment, being near the 15% of occurrences; however, in the suburban area, this value decreases to 5%, probably because also the high-impact ANEs present a lower number of instances. From these results it can be concluded that the detection and removal of ANEs will be more usual in a urban than in a suburban environment, since a significantly higher number of $L_{Aeq,300s}$ values can be biased critically. Furthermore, it is worth mentioning that the number of individual high-impact ANEs may not always coincide with the number of times these events bias the 2 dB threshold. This is because it could happen that two or more high-impact events co-occurred in the same evaluated period of time. However, as shown in the results of this work, this is not the case for the data at hand, thus, all high-impact ANEs occur in different integration times.

The impact patterns observed on both environments present different trends. From the analysis conducted in the suburban area, it was observed that sensor hb104 presents a clearly different pattern of the impact of ANEs compared to the rest of the nodes of that WASN for both week and weekend days. This sensor was installed on a major road with two lanes in each direction with a crossing highway under the bridge (see Table A2), which makes this location substantially different from the other sensors locations in Rome (as they do not correspond to major crossroads). For this sensor, the aggregate ANEs are more likely to bias the $L_{Aeq,300s}$, as a 40% of the analyzed measurements contain a medium or high aggregate impact considering both days. This result leads to the preliminary conclusion that in a suburban area, a crossroad is more susceptible to collect anomalous noise events that may distort the RTN level measurements critically. On the contrary, the data analyzed from the other sensor locations in Rome show that the AIs of the ANEs do not usually have a significant impact on the A-weighted equivalent RTN level measurements. In Milan, however, it becomes difficult to identify specific impact patterns according to the sensor locations due to the great variability of occurrences observed from the recordings of both week and weekend days. Nevertheless, note that all sensors have recorded ANEs with a significant impact—both evaluated individually and in an aggregate manner—being relevant enough to bias the RTN map representation in certain periods of time. Given the fact that the recordings were taken over two days, a relevant number of $L_{Aeq,300s}$ measurements could have been computed with an inaccuracy of more than 2 dB, we can conclude that is necessary to remove all kind of anomalous noise events from the final computation of the noise map.

Briefly, the results drawn from this work present a non-negligible number of anomalous noise events that occur randomly both in the DYNAMAP’s pilot urban and suburban acoustic environments. This is a relevant issue, as we have to mention that the analyzed data correspond only to a recording campaign of two different days, which provide a relevant but limited scope of *all* the possible issues that may occur in all streets and ring road portals during any day of the year at any time. Nevertheless, although the amount of evidence observed in the gathered data may result statistically poor (i.e., only 84 critical pieces of evidence have been observed), their mere presence demonstrates the importance of their automatic removal to obtain reliable dynamic RTN maps through WASN-based approaches. That is, if the sub-sampling done in two days for several 20-min long audio files has led us to this conclusion, what will be the real impact on the measurements in a 24-h × 7-day WASN-based monitoring system? How many works around the city and the highway can occur throughout the year together some horns and sirens? How many sensors can be located close to a school (with the children in the playground) or next to a church with its bells?... This opens a much wider research goal, focused on the detailed analysis of the sensors location and the consequences it entails in terms of anomalous noise events detection and removal, as the election of the sensor’s installation place is usually based on spatial coverage to draw the acoustic map, being also limited by the actual location of the portals and public buildings where the sensors are finally installed.

## 6. Conclusions

In this work, we have analyzed more than 300 h of labeled acoustic data collected through two WASNs after being deployed in the pilot urban and suburban areas of the DYNAMAP project. The study shows that ANEs can be widely found in acoustic environments when monitoring RTN levels in real-operation conditions, being particularly common in the data gathered from the urban area. Moreover, through the impact analysis methodology, it has been also concluded that the aggregate contribution of low and medium-impact ANEs can deviate the $L_{Aeq,300s}$ as critically as high-impact individual ANEs. Therefore, the obtained results highlight the importance of the automatic removal of low, medium and high-impact events to obtain reliable WASN-based RTN maps in real-operation environments.

Future work will be focused on the detailed analysis of the particularities of each acoustic environment and ANEs subcategories together with complex passages, not only to consider their global impact patterns in the urban and suburban, but also to study the spatio-temporal particularities of all the locations and periods of time. Finally, we plan to adapt the preliminary version of the ANED algorithm by using the two WASN-based datasets to improve its performance in both urban and suburban environments in real operations.

## Figures and Tables

**Table 1:** Description and % of occurrences of the 28 sound subcategories attributed to anomalous noise events found throughout the manual labeling process of the WASN-based urban and suburban acoustic databases.

Label	Suburban	Urban	Description
	Counts (%)	Counts (%)	
*airp*	0.1	1	Noise of airplanes and helicopters
*alrm*	0.2	0.3	Sound of an alarm or a vehicle beep moving backwards
*bell*	0	1.2	Church bells
*bike*	<0.1	3.6	Sound of bikes and bike chains
*bird*	15.1	14.7	Birdsong
*blin*	0	<0.1	Opening and closing of a blind
*brak*	23.1	12.7	Brakes and conveyor belts
*busd*	2.8	1.1	Opening bus door (or tramway), depressurized air
*dog*	0	2.5	Barking of dogs
*door*	2.6	14.7	Closing doors (vehicle or house)
*glas*	0	0.1	Sound of glass crashing
*horn*	6.7	3.7	Horns of vehicles (cars, motorbikes, trucks, etc.)
*inte*	0.3	0.2	Interfering signal from an industry or human machine
*musi*	<0.1	0.6	Music in car or in the street
*peop*	0	22.2	Sounds of people chatting, laughing, coughing, sneezing, etc.
*rain*	23.7	0.4	Sound of heavy rain
*rubb*	0	0.1	Rubbish service (engines and grabbing system)
*sire*	1.8	0.7	Sirens (ambulances, police, etc.)
*sqck*	0	0.8	Squeak sound of door hinges
*step*	0	13.7	Sounds of steps
*thun*	7.4	<0.1	Thunderstorm
*trck*	11.9	0	Noise when trucks or vehicles with heavy load passed over a bump.
*tram*	0	0.7	Stop, start and passby sounds of tramways
*tran*	2.7	<0.1	Sound of trains
*trll*	0	1	Sound of wheels of suitcases (trolley)
*stru*	1.4	0	Noise of highway portals structure caused by vibration of trucks passbys
*wind*	0	<0.1	Noise of wind (movement of the leaves of trees,...)
*wrks*	0	4.1	Works in the street (e.g., saws, hammer drills, etc.)

**Table 2:** General characteristics of the WASN-based urban and suburban acoustic databases evaluated considering the impact analysis methodology.

Acoustic Environment	Total Duration	RTN (%)	ANE (%)	CMPLX (%)
Milan (Urban)	151 h	83.7%	8.7%	7.6%
Rome (Suburban)	153 h 20 min	96.5%	1.9%	1.6%

**Table 3:** Number of occurrences and sensor activation ratios per sensor for low, medium and high individual impact ranges.

Individual Impacts		Low Impact		Medium Impact		High Impact	
		(−∞, 0.5) dB		[0.5, 2) dB		[2, +∞) dB	
		**Occurrences**	**Activation**	**Occurrences**	**Activation**	**Occurrences**	**Activation**
		**Count (%)**	**Count/$N_{S}$**	**Count (%)**	**Count/$N_{S}$**	**Count (%)**	**Count/$N_{S}$**
**Milan**	Tuesday	21,264 (99.5%)	23/23	76 (0.4%)	21/23	28 (0.1%)	16/23
	Sunday	15,215 (99.4%)	23/23	58 (0.4%)	20/23	29 (0.2%)	16/23
**Rome**	Tuesday	2105 (98.1%)	19/19	33 (1.6%)	13/19	7 (0.3%)	5/19
	Sunday	3415 (99.0%)	19/19	31 (0.9%)	11/19	5 (0.1%)	3/19

**Table 4:** Number of occurrences and sensor activation ratios per sensor for low, medium and high aggregate impact ranges.

Aggregate Impacts		Low Impact		Medium Impact		High Impact	
		(−∞, 0.5) dB		[0.5, 2) dB		[2, +∞) dB	
		**Occurrences**	**Activation**	**Occurrences**	**Activation**	**Occurrences**	**Activation**
		**Count (%)**	**Count/$N_{S}$**	**Count (%)**	**Count/$N_{S}$**	**Count (%)**	**Count/$N_{S}$**
**Milan**	Tuesday	855 (85.5%)	23/23	107 (10.7%)	22/23	38 (3.8%)	18/23
	Sunday	693 (85.4%)	23/23	88 (10.8%)	21/23	31 (3.8%)	17/23
**Rome**	Tuesday	874 (95.8%)	19/19	29 (3.2%)	12/19	9 (1.0%)	5/19
	Sunday	887 (95.6%)	19/19	35 (3.8%)	13/19	6 (0.6%)	3/19

## Abbreviations

The following abbreviations are used in this manuscript:

AI	Aggregate Impact
ANE	Anomalous Noise Event
ANED	Anomalous Noise Event Detection
CNOSSOS-EU	Common Noise Assessment Methods in Europe
DYNAMAP	Dynamic Noise Mapping
END	European Noise Directive
EU	European Union
RTN	Road Traffic Noise
SNR	Signal-to-Noise Ratio
SONYC	Sounds of New York City
WASN	Wireless Acoustic Sensor Network
WG-AEN	European Commission Working Group Assessment of Exposure to Noise

## Appendix A

This section includes the description of the sensor locations for both urban and suburban environments by means of Table A1 and Table A2, respectively.

**Table A1:** Sensor locations description for the urban environment. X-lane/Y-lane road stands for a two-way road that has X lanes in one sense and Y lanes in the opposite sense. X-lane road stands for a street with X lanes in the same sense.

Sensor Id	Sensor Location Description
**hb106**	1-lane/1-lane road with connection with 1 line road, area with parks nearby, no shops
**hb108**	1-lane/1-lane road, in front University exit, no shops
**hb109**	3-lane/3-lane road, near crossing with tramway and 1 line+2 line/2 line+1 line road, shopping and coffe/restaurant area
**hb115**	1-lane road with shopping in front
**hb116**	1-lane/1-lane road with connection with 1-lane road, residential area
**hb117**	3-lane/3-lane road, near school, area with parks nearby, no shops
**hb120**	1-lane/1-lane road, residential area, no shops
**hb121**	2-lane/2-lane road, connection with 1-lane road, University area, no shops
**hb123**	2-lane/2-lane road with hotel and traffic light nearby
**hb124**	1-lane road, no shops
**hb125**	1-lane road with connection with 1-lane/1-lane road, mix of residential with some shops
**hb127**	1-lane road near bifurcation with 1 line road, some shop nearby
**hb129**	1-lane/1-lane road, bike line, connection with 1-lane road, some shop
**hb133**	1-lane road, residential area, no shops, little park area in front
**hb135**	1-lane road with connection with 1-lane road (low speed), near University campus (students), no shops, in front of park area
**hb136**	1-lane/1-lane road with connection with 1-lane road, area with parks nearby, no shops
**hb137**	1-lane road with connection with 1 line road, in front of park, residential area, no shops
**hb138**	1-lane road near connection with other 1-lane road, no shops
**hb139**	1-lane road, residential area, some shop/enterprise
**hb140**	2-lane/2-lane road with parking area and traffic light with crossing nearby, no shops near and high traffic
**hb144**	1-lane road in residential area, one shop far away
**hb145**	1-lane road, in front of park
**hb151**	1-lane/1-lane road, bike line, some shop and restaurant

**Table A2:** Sensor locations description for suburban environment. X-lane/Y-lane road stands for a two-way road that has X lanes in one sense and Y lanes in the opposite sense. X-lane road stands for a street with X lanes in the same sense.

Sensor Id	Sensor Location Description
**hb103**	Highway with 3-lane/3-lane
**hb104**	Major road with 2-lane each direction crossing a highway under bridge (out of major ring)
**hb105**	Highway with 4-lane (only 1 direction, and near exits/crossings)
**hb110**	Highway with 3-lane/3-lane
**hb111**	Highway with 3-lane/3-lane
**hb112**	Highway with 3-lane/3-lane (near exit and near crossings)
**hb119**	Highway with 3-lane/3-lane
**hb128**	Highway with 3-lane/3-lane
**hb134**	Highway with 4-lane/4-lane (near bridge and crossings)
**hb141**	Highway with 5-lane/5-lane (near crossings)
**hb143**	Highway with 2-lane/2-lane (out of major ring)
**hb147**	Highway with 3-lane/3-lane
**hb148**	Highway with 3-lane/3-lane
**hb149**	Highway with 3-lane (near tunnel)
**hb153**	Major road with 2-lane each direction crossing a highway under bridge (out of major ring)
**hb154**	Highway with 4-lane/4-lane
**hb155**	Highway with 2-lane (near connection but out major ring) plus 1 road same sense next to
**hb156**	Highway with 3-lane/3-lane
**hb157**	Highway with 5-lane/5-lane
